# Dietary Isoacids Improve Early-Life Feed Efficiency, Reduce Abdominal Fat Deposition, and Modulate Intestinal and Metabolic Responses in Broiler Chickens

**DOI:** 10.3390/ani16213478

**Published:** 2026-11-03

**Authors:** Byron Xavier Muñoz, Sara Benavides, Alba Fireman, Cristiano Bortoluzzi, Ivânio José Martins Bueno, Lucas Alves Rodrigues, Raimundo Netto, Mário Lopes, Vitor Pais, Giovana Longhini, Melany Lovon, Cristiane Araujo, Lucio Araujo

**Affiliations:** 1 Department of Animal Science, School of Animal Science and Food Engineering, University of São Paulo, Pirassununga 13635-900, SP, Brazil; sarabenavides@usp.br (S.B.); rnetto@usp.br (R.N.); mhsl.br@usp.br (M.L.); vitorsouza@usp.br (V.P.); giovanamachado@tamu.edu (G.L.); mfernandezl@usp.br (M.L.); lfaraujo@usp.br (L.A.); 2 Zinpro Corporation, Eden Prairie, MN 55344, USA; afireman@zinpro.com (A.F.); cbortoluzzi@zinpro.com (C.B.); ibueno@zinpro.com (I.J.M.B.); lrodrigues@zinpro.com (L.A.R.); 3 Department of Animal Production and Nutrition, School of Veterinary Medicine and Animal Science, University of São Paulo, Pirassununga 13635-900, SP, Brazil; crsilva@usp.br

**Keywords:** broiler, cecal microbiome, isoacids, metabolomic, performance

## Abstract

This study evaluated the effects of increasing dietary inclusion levels of branched-chain volatile fatty acids, also called isoacids (ISO), on the growth performance, carcass traits, intestinal health, and breast muscle metabolomic profile of broiler chickens. A total of 700 one-day-old male Cobb 500 chicks were assigned to five corn–soybean meal-based diets with 10 replicate pens (14 birds/pen) in a completely randomized design: A Positive Control (PC) diet formulated to meet nutritional requirements was supplemented with 0.0, 0.10, 0.20, 0.40, or 0.60% of ISO (supplied as a blend of isobutyrate and 2-methylbutyrate). Data were analyzed using General Linear Models with orthogonal polynomial contrasts, PERMANOVA for microbiome beta diversity and ANOVA with FDR correction for metabolomics. Growth performance was monitored across feeding phases. At day 42, carcass traits, breast meat quality, intestinal permeability, cecal short-chain fatty acids (SCFAs), and cecal and ileal microbiota were assessed, alongside liquid chromatography coupled to high-resolution mass spectrometry (LC-HRMS) metabolomics of the Pectoralis major muscle. Dietary ISO significantly improved average daily weight gain (*p* < 0.0001) and feed conversion ratio (*p* < 0.0001) during the first 21 days (early-life), with an optimal inclusion level of 0.40%. While overall carcass and breast yields were unaffected, ISO supplementation significantly and quadratically reduced abdominal fat deposition (*p* < 0.0001), with an optimal inclusion range between 0.10% and 0.40%. ISO did not compromise intestinal permeability or alter microbial alpha diversity, but it significantly restructured the cecal microbiota (beta diversity; Bray–Curtis *p* = 0.0213) and linearly increased cecal isobutyric acid accumulation in the cecal content (*p* = 0.0443). Breast muscle metabolomics revealed a dose-dependent, nonlinear response; the 0.40% inclusion level maximized the relative abundance of galactolipids, whereas the highest dose (0.60% ISO) upregulated sphingomyelins, lysophospholipids, and acylcarnitines. In conclusion, a specific dietary ISO supplementation dose of 0.4% serves as an effective nutritional strategy to boost early feed efficiency and reduce abdominal adiposity. Doses exceeding this optimal level, such as 0.60%, offer no further productive benefits and may trigger cellular stress.

## Simple Summary

In poultry production, finding sustainable feed additives that improve bird growth and health without relying on antibiotics is essential. Isoacids (ISO) are natural compounds produced by gut bacteria that have proven effective in cattle, but their effects on broiler chickens remain largely unknown. This study evaluated how adding different levels of dietary ISO affects growth, meat quality, gut health, and muscle metabolomics of broiler chickens over a 42-day period. Our findings revealed that chickens fed 0.4% of ISO significantly improved early-life feed efficiency. Furthermore, the supplement significantly reduced unwanted abdominal fat accumulation, without affecting overall meat yields. Although the highest dose (0.6%) upregulated specific metabolites such as lysophospholipids and acylcarnitines in the muscle, the 0.4% level maximized the relative abundance of galactolipids and preserved intestinal microbial richness. This research provides valuable insights for the poultry industry, demonstrating that, when supplemented within this optimal range, dietary ISO serves as an effective nutritional strategy to enhance production efficiency and carcass quality, ultimately contributing to more sustainable and cost-effective meat production for society.

## 1. Introduction

Branched-chain volatile fatty acids (BCVFAs), commonly referred to as isoacids (ISO), are microbial metabolites generated during the anaerobic degradation of the branched-chain amino acids (BCAAs) valine, leucine, and isoleucine, yielding isobutyrate, isovalerate, and 2-methylbutyrate, respectively [1,2]. In ruminants, these compounds have been extensively investigated because they serve as metabolic intermediates and growth factors for specific ruminal microorganisms, particularly fibrolytic populations involved in structural carbohydrate digestion. Supplementation of ISO has been associated with improved microbial protein synthesis, fiber degradation, and nutrient utilization efficiency [2].

In poultry, however, microbial fermentation is largely confined to the ceca, where dense microbial communities metabolize substrates escaping digestion in the upper gastrointestinal tract (GIT), including resistant carbohydrates and undigested protein fractions [3,4]. This hindgut ecosystem contributes to host nutrition through the production of microbial metabolites that can affect intestinal physiology, epithelial function, and immune responses. Because BCVFAs are primarily derived from amino acid fermentation, their production in broilers is expected to depend on dietary protein level, amino acid digestibility, digesta retention time, and cecal microbiota composition.

Most studies evaluating microbial metabolites in broiler chickens have focused on short-chain fatty acids (SCFAs), especially acetate, propionate, and butyrate. These compounds are recognized for their beneficial effects on intestinal health, including provision of energy to enterocytes, modulation of inflammatory responses, improvement of barrier integrity, and suppression of enteric pathogens [5]. Butyrate supplementation has also been associated with improved growth performance and intestinal morphology in broilers under several production conditions. In contrast, the physiological significance of BCVFAs in poultry remains poorly characterized [6].

Evidence from other monogastric models indicates that metabolites originating from protein fermentation may influence epithelial metabolism, nitrogen recycling, and microbial ecology, suggesting biological functions beyond their traditional classification as terminal products of amino acid catabolism [7,8]. Nevertheless, controlled in vivo studies evaluating dietary ISO supplementation in broiler chickens are scarce or absent. This lack of information is particularly relevant given the growing interest in nutritional strategies capable of improving feed efficiency, gut health, and production sustainability without relying on antimicrobial growth promoters.

Despite their documented biological benefits in poultry nutrition, the dietary supplementation of volatile and organic acids must be strictly dose-regulated. Previous research in broiler chickens has demonstrated that excessive dietary inclusion levels of propionate can exert adverse effects, significantly depress voluntary feed intake and impair overall growth performance [9,10]. When ingested in high concentrations, these organic compounds overwhelm the animal’s natural buffering capacity, profoundly altering the acid–base equilibrium in the bloodstream. This disruption inevitably triggers metabolic acidosis [11]. Establishing an optimal dietary inclusion threshold is critical to maximize productive advantages while preventing performance depression and metabolic toxicity.

The present study aimed to evaluate the effects of dietary inclusion levels of ISO on growth performance, carcass characteristics, intestinal permeability, cecal microbiota, and breast meat metabolomic profile of broiler chickens.

## 2. Materials and Methods

### 2.1. Ethical Approval and Experimental Facilities

The trial was carried out in a commercial-type broiler research facility of the Department of Animal Science, School of Animal Science and Food Engineering (FZEA), University of São Paulo (USP), located at the Fernando Costa campus in Pirassununga, São Paulo. All experimental procedures involving animals were approved by the Animal Care and Use Ethics Committee of the School of Animal Science and Food Engineering, University of São Paulo (protocol no. 7495230625).

### 2.2. Birds, Experimental Design, and Husbandry

A total of 700 one-day-old male Cobb 500 broiler chicks were randomly allocated, in 50 floor pens (1.00 × 1.2 m^2^) with 14 birds per pen (11.67 birds/m^2^ at placement and 42 kg/m^2^ at 42 days), to one of five dietary treatments in a completely randomized design, considering each pen as the experimental unit with ten replicate pens per treatment. Each pen was bedded with fresh wood shavings and equipped with nipple drinkers and a tube feeder. Birds had ad libitum access to feed and water during the entire experiment. Lighting and temperature programs were adjusted according to breeder management recommendations for Cobb 500 broilers. Chicks received their complete vaccination program at the commercial hatchery before being transported to the experimental facility, following standard commercial procedures. No further vaccines, antibiotics, or medications were provided during the experimental period. The brooding temperature was initially maintained at 33 °C and progressively lowered to 20 °C by 28 days of age to maintain animal comfort. Stocking density was maintained within commercially relevant limits until 42 d of age [12].

### 2.3. Experimental Diets

Corn–soybean meal-based diets containing meat and bone meal were formulated for three feeding phases: starter (1–21 d), grower (22–35 d), and finisher (36–42 d). The complete formulation and nutrient composition of the experimental diets are shown in Table 1. A positive control (PC—0%) diet was formulated to meet nutrient requirements according to Brazilian Tables for Poultry and Swine [13]. The PC diet was supplemented with increasing inclusion levels of ISO (0.0, 0.10, 0.20, 0.40, or 0.60%). The additive consisted of a 2:1 blend of isobutyrate and 2-methylbutyrate (Zinpro Corp., Eden Prairie, MN, USA). Feed was offered in mash form.

### 2.4. Growth Performance

Body weight and feed intake were recorded by pen at 7, 21, 35 and 42 d. Variables calculated included body weight gain (BWG), daily feed intake (DFI), feed conversion ratio (FCR), daily weight gain (DWG), and final body weight (FBW). Mortality was recorded twice daily, and performance variables were corrected for mortality when appropriate using the standard industry formula: FCR adjusted = Total Feed Intake per Pen (g)/[Weight Gain of Surviving Birds (g) + Weight Gain of Dead Birds prior to removal (g)].

### 2.5. Slaughter Procedures and Carcass Yield

At 42 d, three birds from each pen (representative of the pen’s average body weight) were selected for processing. After feed withdrawal (10 h), birds were transported to the university processing plant, electrically stunned, exsanguinated, scalded, defeathered, and manually eviscerated under commercial conditions. Chilled carcasses were deboned, and breast meat, wings, and leg quarters were weighed. Yields were expressed relative to chilled carcass weight according to the strain’s guide [14].

### 2.6. Breast Meat Quality

One breast fillet (Pectoralis major) from one bird from each of six randomly selected pens per treatment was evaluated 24 h postmortem for proximate composition, pH, and physical quality traits. Ultimate meat pH was measured directly on the fillet using a digital penetrating pH meter, while objective color coordinates (L*, a*, b*) were recorded using a Minolta colorimeter CR400 (Osaka, Japan) according to CIE standards. Warner–Bratzler shear force was used to determine the tenderness of cooked samples, and cooking loss was determined following standard procedures [15]. Finally, lipid peroxidation was evaluated through the thiobarbituric acid reactive substances (TBARS) assay by measuring malondialdehyde (MDA) content spectrophotometrically at 532 nm, with results expressed as mg MDA/kg of meat [16].

### 2.7. Intestinal Microbiome Analysis

At 42 d, ileal and cecal digesta samples from one bird from each of six randomly selected pens per treatment were aseptically collected, snap-frozen in liquid nitrogen, and stored at −80 °C. DNA was extracted using a commercial kit QIAamp PowerFecal Pro DNA Kit (QIAGEN, Hilden, Germany). Bacterial communities were characterized by sequencing the V3–V4 region of the 16S rRNA gene. Libraries were prepared and sequenced using the Illumina MiSeq platform (2 × 300 bp) (Illumina Inc., San Diego, CA, USA) [17]. Bioinformatic processing was performed in QIIME2, including denoising, chimera removal, amplicon sequence variant (ASV) generation, and taxonomic assignment. Similar sequencing strategies have been widely applied in broiler microbiome studies [18].

**Table 1:** Composition of experimental diets supplemented with isoacids for commercial male broilers.

	Starter (1–21 d)					Grower (22–35 d)					Finisher (35–42 d)				
Inclusion of ISO (%)	0.0	0.1	0.2	0.4	0.6	0.0	0.1	0.2	0.4	0.6	0.0	0.1	0.2	0.4	0.6
Ingredient (% as Feed)															
Corn	55.81	55.62	55.43	55.05	54.67	59.66	60.90	62.15	64.63	64.91	63.22	64.46	65.70	67.83	67.73
Soybean meal, 46%	37.14	37.16	37.18	37.23	37.28	34.15	32.99	31.83	29.51	29.17	30.21	29.05	27.89	25.89	25.90
Soybean oil	1.13	1.19	1.26	1.39	1.52	1.55	1.29	1.03	0.50	0.36	2.51	2.25	1.98	1.52	1.45
Limestone	0.67	0.67	0.66	0.66	0.66	0.70	0.70	0.70	0.70	0.70	0.71	0.71	0.71	0.71	0.71
Bone and meat meal	3.60	3.60	3.60	3.61	3.61	2.42	2.43	2.45	2.49	2.50	1.88	1.89	1.91	1.94	1.95
Salt (NaCl)	0.42	0.42	0.42	0.42	0.42	0.37	0.37	0.37	0.37	0.37	0.35	0.35	0.35	0.35	0.35
Lys-HCl, 78.8%	0.22	0.22	0.22	0.22	0.22	0.20	0.23	0.27	0.33	0.34	0.20	0.23	0.27	0.32	0.32
DL- Met, 99%	0.35	0.35	0.35	0.35	0.35	0.31	0.32	0.33	0.35	0.35	0.28	0.29	0.30	0.31	0.31
L-Thr, 98%	0.21	0.21	0.21	0.21	0.21	0.19	0.19	0.18	0.17	0.14	0.18	0.18	0.17	0.16	0.12
Choline chloride 60%	0.06	0.06	0.06	0.06	0.06	0.05	0.05	0.05	0.06	0.06	0.05	0.05	0.05	0.06	0.06
Salinomycin	0.03	0.03	0.03	0.03	0.03	0.03	0.03	0.03	0.03	0.03	0.03	0.03	0.03	0.03	0.03
Phytase	0.02	0.02	0.02	0.02	0.02	0.02	0.02	0.02	0.02	0.02	0.02	0.02	0.02	0.02	0.02
Xylanase	0.01	0.01	0.01	0.01	0.01	0.01	0.01	0.01	0.01	0.01	0.01	0.01	0.01	0.01	0.01
Isoacids	0.00	0.10	0.20	0.40	0.60	0.00	0.10	0.20	0.40	0.60	0.00	0.10	0.20	0.40	0.60
Vitamin premix ^1^	0.05	0.05	0.05	0.05	0.05	0.05	0.05	0.05	0.05	0.05	0.05	0.05	0.05	0.05	0.05
Mineral premix ^2^	0.10	0.10	0.10	0.10	0.10	0.10	0.10	0.10	0.10	0.10	0.10	0.10	0.10	0.10	0.10
L-Valine, 96.5%	0.20	0.20	0.20	0.19	0.19	0.21	0.23	0.25	0.29	0.30	0.22	0.24	0.26	0.30	0.30
Total	100	100	100	100	100	100	100	100	100	100	100	100	100	100	100
Analyzed composition															
Crude protein (%)	21.67	19.48	22.2	22.71	25.19	20.45	20.86	22.5	20.98	21.24	19.22	19.61	21.15	19.72	19.97
Moisture (%)	11.49	11.54	11.56	11.21	11.09	11.84	11.46	11.5	11.42	11.64	12.21	11.62	11.86	11.77	12.00
Ether extract (crude fat, %)	4.48	4.62	5.32	5.22	5.11	4.68	4.53	4.75	4.96	5.19	4.54	4.29	4.31	4.81	5.03
Crude fiber (%)	4.01	3.81	3.54	4.19	4.56	2.91	4.01	3.69	3.75	3.15	3.52	3.71	3.42	3.28	2.94
Ash (%)	5.97	5.54	5.54	6.42	7.01	5.64	6.14	6.48	6.14	6.19	5.78	6.30	6.65	6.30	6.35

^1^ Vitamins provided per kg of premix: vitamin A, 13,227,513 IU; vitamin D3, 3,968,253 IU; vitamin E, 66,137 IU; vitamin B12, 39.6 mg; riboflavin, 13,227 mg; niacin, 110,229 mg; d-pantothenic acid, 22,045 mg; menadione, 3968 mg; folic acid, 2204 mg; vitamin B6, 7936 mg; thiamine, 3968 mg; biotin, 253.5 mg. ^2^ Trace minerals provided per kg of premix: manganese (Mn SO4), 60 g; zinc (ZnSO4), 60 g; iron (FeSO4), 40 g; copper (CuSO4), 5 g; iodine (Ca(IO3)2), 1.25 g.

### 2.8. Cecal Short-Chain Fatty Acids

The cecal SCFA profile (acetate, propionate, isobutyrate, butyrate, isovalerate, and valerate) was determined by GC-FID, adapting the method described by Erwin et al. [19]. Cecal samples from one bird from each of six randomly selected pens per treatment were diluted in distilled water (1:2 *w*/*v*), homogenized, and centrifuged at 3500 rpm for 10 min. Subsequently, 0.8 mL of the supernatant was mixed with 0.2 mL of formic acid (HPLC grade) and 0.1 mL of 2-ethylbutyric acid (internal standard, 100 mM), followed by a second centrifugation at 11,000 rpm for 8 min before being transferred to a chromatography vial. Analysis was performed on a Shimadzu gas chromatograph (GCSolution software V 2.44) equipped with a Stabilwax^®^ column (30 m × 0.53 mm i.d.) operated isothermally at 140 °C, with injector and detector temperatures set at 250 °C. Helium was used as the carrier gas (column flow: 7.5 mL/min). The injection volume was 1 µL with a total run time of 8 min. Quantification was performed using external calibration curves interpolated with the internal standard and adjusted for the initial sample weight.

### 2.9. Intestinal Permeability

Gut barrier function was assessed using fluorescein isothiocyanate dextran (FITC-dextran; 3–4 kDa; Sigma-Aldrich, St. Louis, MO, USA). One bird from each of eight randomly selected pens per treatment was orally gavaged with FITC-dextran after a short fasting period. Blood samples were collected 2.5 h later, plasma was separated, and fluorescence intensity was measured using a microplate reader (excitation 485 nm; emission 528 nm). Plasma FITC-dextran concentration was calculated from standard curves, with higher values interpreted as increased intestinal permeability [20].

### 2.10. Breast Muscle Metabolomics

At day 42, immediately following exsanguination and evisceration, breast meat tissue samples (Pectoralis major) were harvested from one bird from each of six randomly selected pens per treatment. The sampled birds were carefully selected within ±5% of the average pen body weight to ensure uniformity, confirming 6 independent biological replicates per treatment. Tissue samples were snap-frozen in liquid nitrogen within 10 min post-mortem to prevent enzymatic metabolite degradation and stored at −80 °C until analytical extraction. For the untargeted metabolomic profiling, approximately 0.5 g of frozen breast muscle was precisely weighed and sectioned while maintained on dry ice. The tissue was transferred to bead beating homogenization tubes containing precooled stainless-steel beads. Cellular disruption was performed via a mechanical homogenizer at 4 °C using a monophasic extraction solvent mixture of ice-cold aqueous methanol to disrupt lipid protein complexes and solubilize low molecular weight polar metabolites alongside intracellular lipid fractions [21].

Following complete homogenization, the crude extract was incubated on ice for 30 min to facilitate complete protein precipitation and phase equilibrium. The mixture was then clarified via ultra centrifugation at 10,000× *g* for 15 min at 4 °C to yield a solid macromolecular pellet and a translucent supernatant. The resulting supernatant, containing the enriched metabolome, was collected, transferred to clean amber vials, and completely lyophilized under vacuum. Prior to liquid chromatography coupled to high resolution mass spectrometry (LC–HRMS) analysis, the dried metabolic residues were reconstituted in a mass-spectrometry-grade solvent matrix, centrifuged to remove any remaining particulates, and transferred to autoinjector vials.

The reconstituted extracts were analyzed via an LC-HRMS system. The raw mass spectra were systematically processed for automated peak detection, deconvolution, and retention time alignment using Compound Discoverer™ 3.3 software (Thermo Fisher Scientific, Waltham, MA, USA) [22]. Metabolite annotation and chemical identity validation were conducted based on accurate mass retention profiles (MS1), characteristic isotope patterns, and specific tandem mass spectrometry (MS/MS) fragmentation patterns against the high-resolution reference datasets within the mzCloud and ChemSpider databases.

### 2.11. Statistical Analysis

Normality and homogeneity of variances were assessed using the Shapiro–Wilk and Levene’s tests, respectively. Data were analyzed using the General Linear Model (GLM) or Mixed procedures of SAS software (version 9.4; SAS Institute Inc., Cary, NC, USA), strictly maintaining the pen as the experimental unit across all analyses. Variables measured directly at the pen level (e.g., growth performance) and variables where a single bird was sampled from randomly selected pens (e.g., meat quality, intestinal permeability, and cecal SCFAs; *n* = 6 or 8 pens/treatment) were analyzed using a one-way ANOVA with dietary treatment as the sole fixed effect. For parameters where multiple birds were sampled per pen (i.e., carcass traits, 3 birds/pen), data were analyzed using a mixed-model ANOVA incorporating dietary treatment as a fixed effect and pen nested within treatment as a random effect to account for observational dependence and hierarchical structure. Regression analysis using orthogonal polynomial contrasts were used to evaluate linear and quadratic responses to increasing dietary ISO inclusion levels, and treatment means were compared using Tukey’s test (*p* ≤ 0.05). For microbiome analysis, alpha diversity was assessed using Chao1, Fisher, and Shannon indices, whereas beta diversity was evaluated using Bray–Curtis, Jaccard, weighted UniFrac, and unweighted UniFrac distances, with treatment effects tested by permutational multivariate analysis of variance PERMANOVA. Differential abundance analysis was performed for taxa with mean relative abundance > 2% using Kruskal–Wallis and Dunn’s post hoc tests (*p* ≤ 0.05). Taxonomic assignment was performed using the Genome Taxonomy Database (GTDB) [23]. For metabolomic data, individual peak areas were normalized by total ion intensity to correct for matrix effects and analytical variance, followed by log_10_-transformation. The normalized dataset was submitted to one-way ANOVA within the MetaboAnalyst 5.0 framework [24] and R software (v4.3) to evaluate the metabolic effects of dietary ISO inclusion levels. Multiple comparisons were adjusted using the Benjamini–Hochberg false discovery rate (FDR) correction. The top 15 highly discriminant breast muscle metabolites with the lowest FDR values (FDR < 0.05) were selected for exploratory multivariate projection using Partial Least Squares Discriminant Analysis (PLS-DA) and hierarchical clustering visualization using Euclidean distance metrics and Ward’s linkage method.

## 3. Results

### 3.1. Growth Performance

The growth performance responses of broiler chickens, including average daily weight gain (ADWG), average daily feed intake (ADFI), and feed conversion ratio (FCR) across all feeding phases, are summarized in Table 2. During the 1 to 7 d phase, dietary ISO supplementation significantly influenced ADWG (*p* = 0.0067), which exhibited a significant positive linear response (*p* = 0.0048) to increasing inclusion levels, where broilers fed 0.4% ISO achieved the highest gain (21.80 g). ADFI was not significantly affected by the treatments (*p* = 0.3143) and showed no distinct regression trends during this initial week. Conversely, FCR was markedly improved by the dietary treatments (*p* = 0.0006), displaying a highly significant linear reduction (*p* < 0.0001) as ISO levels increased, with the lowest conversion ratios observed at the 0.4% (1.024 g/g) and 0.6% (1.019 g/g) inclusion levels. In the 1 to 21 d phase, dietary treatments had a highly significant effect on ADWG (*p* < 0.0001). This variable responded in a linear manner (*p* < 0.0001), with the greatest ADWG observed in birds receiving 0.2% (50.65 g) and 0.4% (51.72 g) ISO. ADFI was also significantly altered (*p* = 0.0012) and followed a quadratic regression trend (*p* = 0.0031), where feed consumption peaked at 0.4% inclusion (62.06 g) before declining at the highest dose. Concurrently, FCR was significantly enhanced (*p* < 0.0001) and demonstrated a linear decrease (*p* = 0.0453) relative to the control group, reaching its optimal values between 0.2% and 0.6% inclusion. During the 1 to 35 d phase, the overall treatment effects did not reach full statistical significance for ADWG (*p* = 0.0576) or ADFI (*p* = 0.0681). However, regression analysis revealed significant positive linear trends for both variables (*p* = 0.0042 for ADWG; *p* = 0.0169 for ADFI), indicating a steady increase in daily gain and feed consumption as dietary ISO increased, with the highest numerical values for both parameters recorded at 0.4% inclusion (69.88 g for ADWG and 98.95 g for ADFI). FCR was not significantly influenced by the treatments (*p* = 0.0814) during this period. Finally, in the 1 to 42 d phase, dietary ISO supplementation did not significantly modify any of the evaluated performance traits (*p* > 0.05). ADWG (*p* = 0.9060), ADFI (*p* = 0.3939), and FCR (*p* = 0.3980) remained statistically uniform across all experimental groups, and no significant linear or quadratic regression trends were detected.

### 3.2. Carcass and Cut-Up Yields

Dietary ISO supplementation did not significantly alter carcass yield (*p* = 0.5339), breast meat percentage (*p* = 0.2052), or wing proportion (*p* = 0.2751) (Table 3). Legs yield exhibited a significant negative linear response (*p* = 0.0111) as dietary ISO levels increased, with a gradual reduction across treatments. Abdominal fat deposition was significantly affected (*p* < 0.0001), showing a quadratic response. Dietary ISO inclusion successfully downregulated adipose accumulation, with all supplemented cohorts displaying markedly lower abdominal fat percentages (*p* < 0.05) than the 0.0% birds (1.57%). Based on the quadratic regression model (y = 1.44 − 2.47x + 3.15x^2^; R^2^ = 0.5993), the mathematical minimum for abdominal fat deposition was estimated at 0.39%. However, given the substantial similarity in fat reduction across all supplemented groups compared to the control, this vertex represents an approximate threshold within a broader effective response range (0.10% to 0.40%) where lipid accumulation establishes a well-defined plateau.

### 3.3. Breast Meat Quality Evaluation

Dietary ISO supplementation did not significantly alter the physical meat quality attributes of broiler breast muscle (*p* > 0.05). Shear force (averaging 20.7 N) and cooking loss (21.4%) remained statistically uniform and unaffected by the additive concentrations. Objective meat color coordinates were similarly stable, maintaining overall baseline means of 56.2 for lightness (L*), 6.2 for redness (a*), and 11.4 for yellowness (b*), with only a minor, nonsignificant numerical decline in the a* coordinate observed at the maximum 0.6% inclusion level. Conversely, while the overall treatment effect for lipid peroxidation index showed a nonsignificant trend (*p* = 0.088), TBARS values exhibited a significant positive linear regression (*p* = 0.011). This linear progression was characterized by a steady increase in MDA content as ISO levels increased, increasing from a baseline of 0.533 mg MDA/kg in the 0.0% treatment to peak concentrations of 1.067 mg MDA/kg and 1.050 mg MDA/kg in the 0.2% and 0.4% groups, respectively.

### 3.4. Intestinal Microbiome

#### 3.4.1. Microbial Diversity

**Figure 1:**
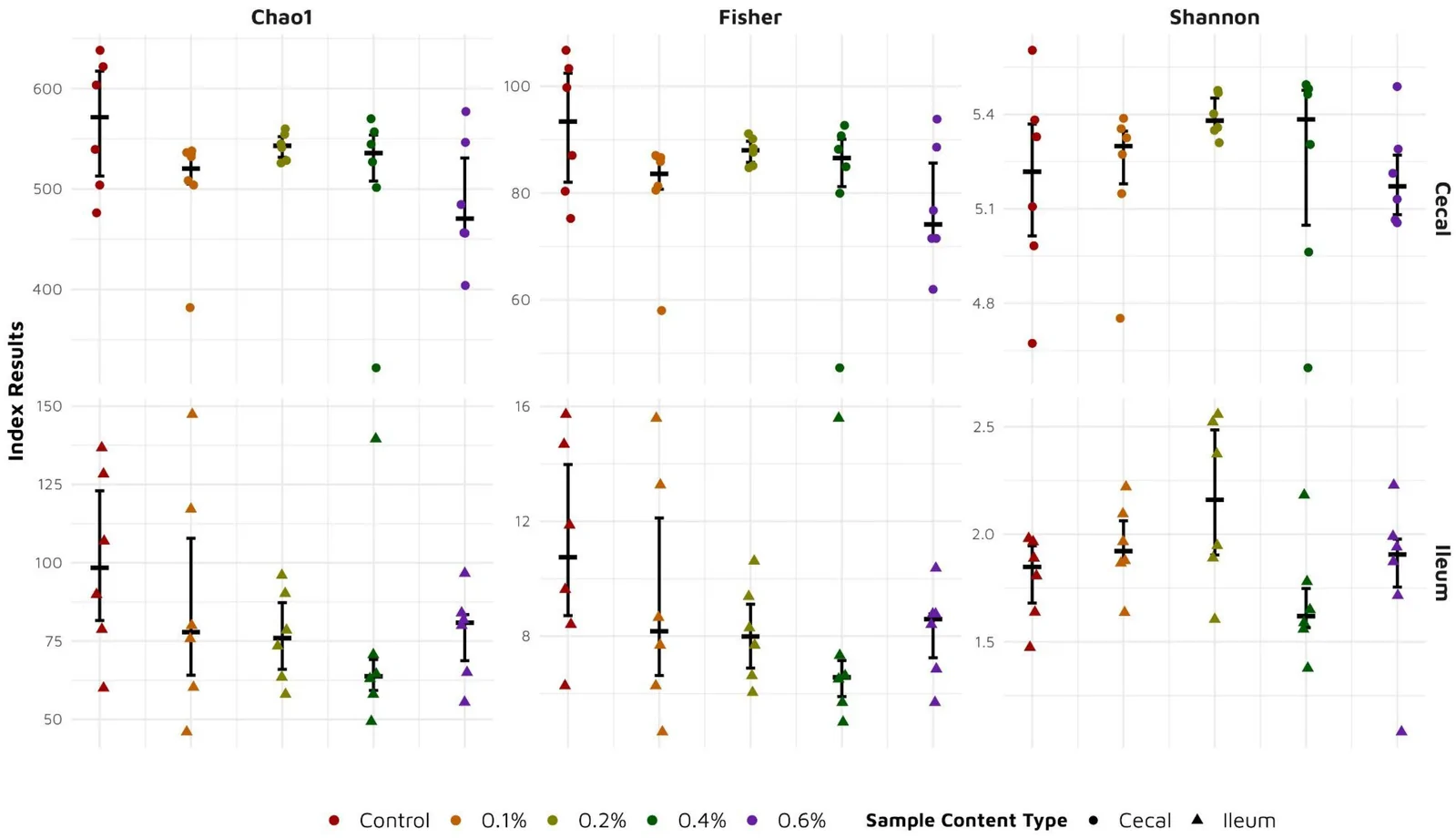
Effect of isoacids on Alpha diversity analysis in cecum and ileum using Chao 1, Fisher and Shannon indexes of commercial male broilers. Vertical lines indicate Q1 and Q3 extension, and the horizontal line represents the median.

**Figure 2:**
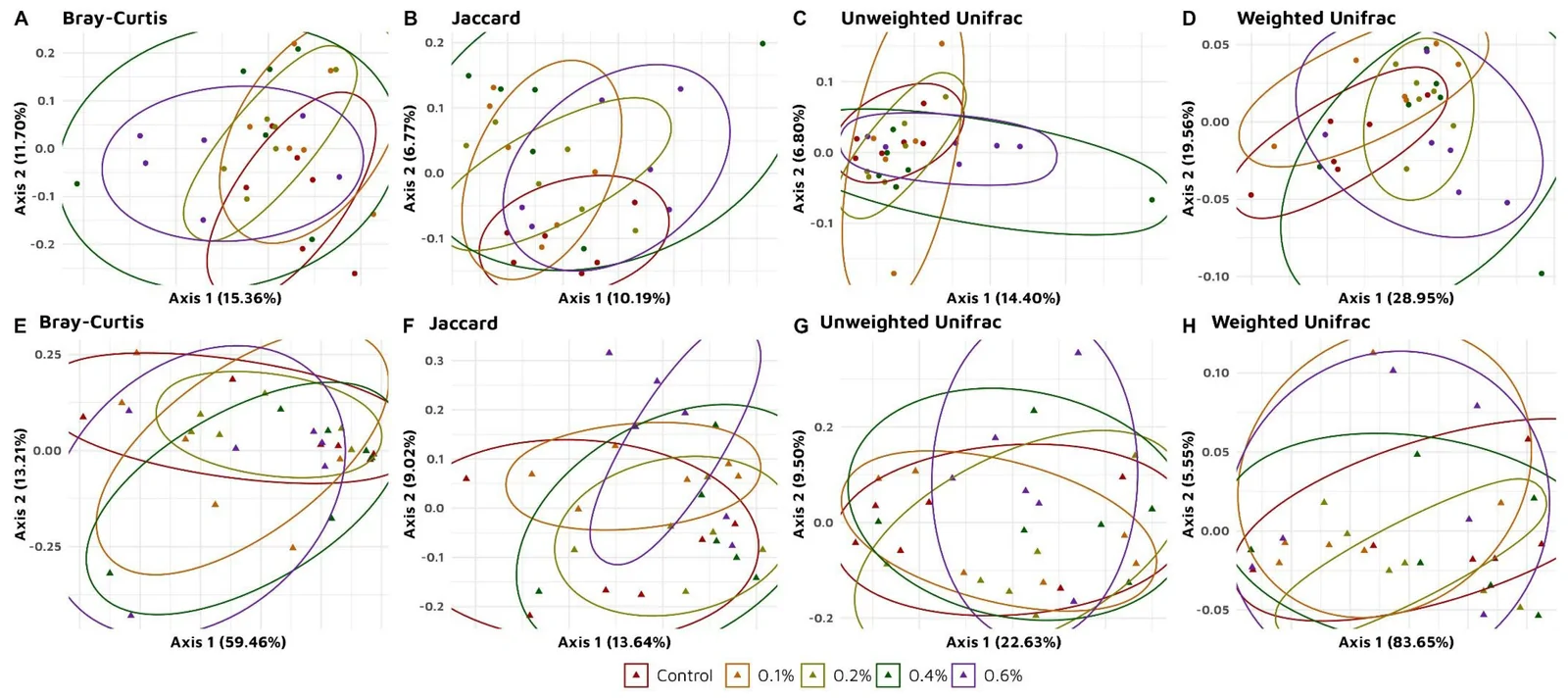
Effect of isoacids on Beta diversity analysis in cecum and ileum of commercial male broilers.

Although dietary ISO supplementation did not statistically alter alpha diversity metrics (*p* > 0.05; Figure 1), distinct, compartment-specific visual trends emerged. The cecum consistently maintained a higher baseline of richness and diversity than the ileum. In the cecal environment, intermediate ISO doses (0.2% and 0.4%) stabilized the community by reducing individual sample dispersion and slightly increasing evenness (Shannon index), whereas the 0.6% inclusion level inverted this effect, reducing richness and increasing variance. In the ileum, a nonlinear pattern was apparent: 0.2% ISO slightly elevated the Shannon index median, while 0.4% marked a sharp decline in overall metrics before returning to baseline configurations at 0.6%. Beta diversity analyses uncovered distinct, tissue specific mechanisms shaping the microbial community structures (Table 4; Figure 2). The cecal microbiota underwent a significant overall restructuring across quantitative and qualitative matrices, including Bray–Curtis (R^2^ = 0.1713, *p* = 0.0213), Jaccard (R^2^ = 0.1567, *p* = 0.0161), and Weighted UniFrac (R^2^ = 0.1138, *p* = 0.0312). Pairwise comparisons confirmed that lower doses (0.1% and 0.2%) diverged from the control group under Bray–Curtis, while Jaccard distances separated the control from the 0.1% and 0.4% groups. Furthermore, Weighted UniFrac metrics demonstrated that the 0.6% dose established a distinct structural profile from both the control and 0.2% cohorts. Conversely, the ileal microbiota shifted through an exclusively qualitative mechanism, modifying community membership rather than abundance. Significant variations were restricted to the Jaccard (R^2^ = 0.1715, *p* = 0.0104) and Unweighted UniFrac (R^2^ = 0.1677, *p* = 0.0145) indices, while Bray–Curtis and Weighted UniFrac, showed extensive spatial overlapping (*p* > 0.05). This ileal shift was primarily driven by the 0.6% inclusion level, which pairwise Jaccard comparisons discriminated from the control, 0.1%, and 0.2% groups, indicating that the maximum dosage altered core ASV membership and lineages while preserving dominant local taxa.

#### 3.4.2. Bacterial Community

The predominant phyla across both digestive segments were *Bacillota* (formerly Firmicutes) and Bacteroidota. At the class level, Clostridia, Bacilli, and Bacteroidia were the most abundant cohorts in both sample types. The dominant orders identified were Peptostreptococcales, Lactobacillales, Oscillospirales, Lachnospirales, MOL361, TANB77, Bacteroidales, and Christensenellales. At the family level, Peptostreptococcaceae, Lactobacillaceae, Lachnospiraceae, Ruminococcaceae, Oscillospiraceae, Turicibacteraceae, CAG-508, and Acutalibacteraceae exhibited the highest relative abundances across all communities. The most abundant genera comprised *Romboutsia*, *Lactobacillus*, *Faecalibacterium*, *Turicibacter*, *Ligilactobacillus*, *Mediterraneibacter*, and *Limosilactobacillus*. Correspondingly, the dominant species across both cecal and ileal contents were *Romboutsia* sp08336475, *Turicibacter bilis*, *Faecalibacterium* sp0021608D5, *Mediterraneibacter ornithocaccae*, and *Ligilactobacillus agilis*. The individual assessment of the Bacillota/Bacteroidota (F/B) ratio (ST13 and ST14) revealed no statistically significant variations (*p* > 0.05) among the dietary ISO inclusion levels. Hierarchical clustering via heatmap analysis at the genus level did not demonstrate clear divergence patterns attributable to ISO concentrations; instead, samples clustered predominantly by anatomical segment origin.

#### 3.4.3. Differential Abundance of Taxa

In cecal samples, the families Acutalibacteraceae, Lachnospiraceae, Peptostreptococcaceae, and Turicibacteraceae displayed significant variations under the Kruskal–Wallis test. However, subsequent Dunn’s post hoc tests confirmed significant pairwise differences solely within the Lachnospiraceae family, distinguishing the 0.1% from the 0.2% ISO treatments. At the genus level, *Romboutsia* and *Turicibacter* showed significant differences under the initial Kruskal–Wallis screening in cecal samples, but these variations did not resolve into statistical significance during Dunn’s pairwise post hoc comparisons. Similarly, *Romboutsia* sp018336475 was the only species displaying overall significance via the Kruskal–Wallis test in the cecum, though no specific intertreatment differences were validated by the post hoc analysis.

### 3.5. Cecal Short-Chain Fatty Acids

Dietary ISO supplementation did not significantly alter the baseline cecal fermentation profiles of major short-chain fatty acids (Table 5). Acetic acid remained the predominant profile across all groups, accounting for 70% to 74% of the total SCFA molar ratio (*p* = 0.4051), while propionic (*p* = 0.3459), butyric (*p* = 0.4929), isovaleric (*p* = 0.3329), and valeric (*p* = 0.5364) acids remained statistically uniform and within narrow biological ranges. Conversely, a distinct metabolic shift was observed for the branched-chain fraction; while the overall treatment effect for isobutyric acid was not significant (*p* = 0.1650), regression analysis revealed a marginally significant linear trend (*p* = 0.0443). Isobutyric acid numerically increased from 1.06% in the 0.0% group to a peak of 1.55% at the 0.4% inclusion level, before slightly declining at the highest dose.

### 3.6. Intestinal Permeability

**Figure 3:**
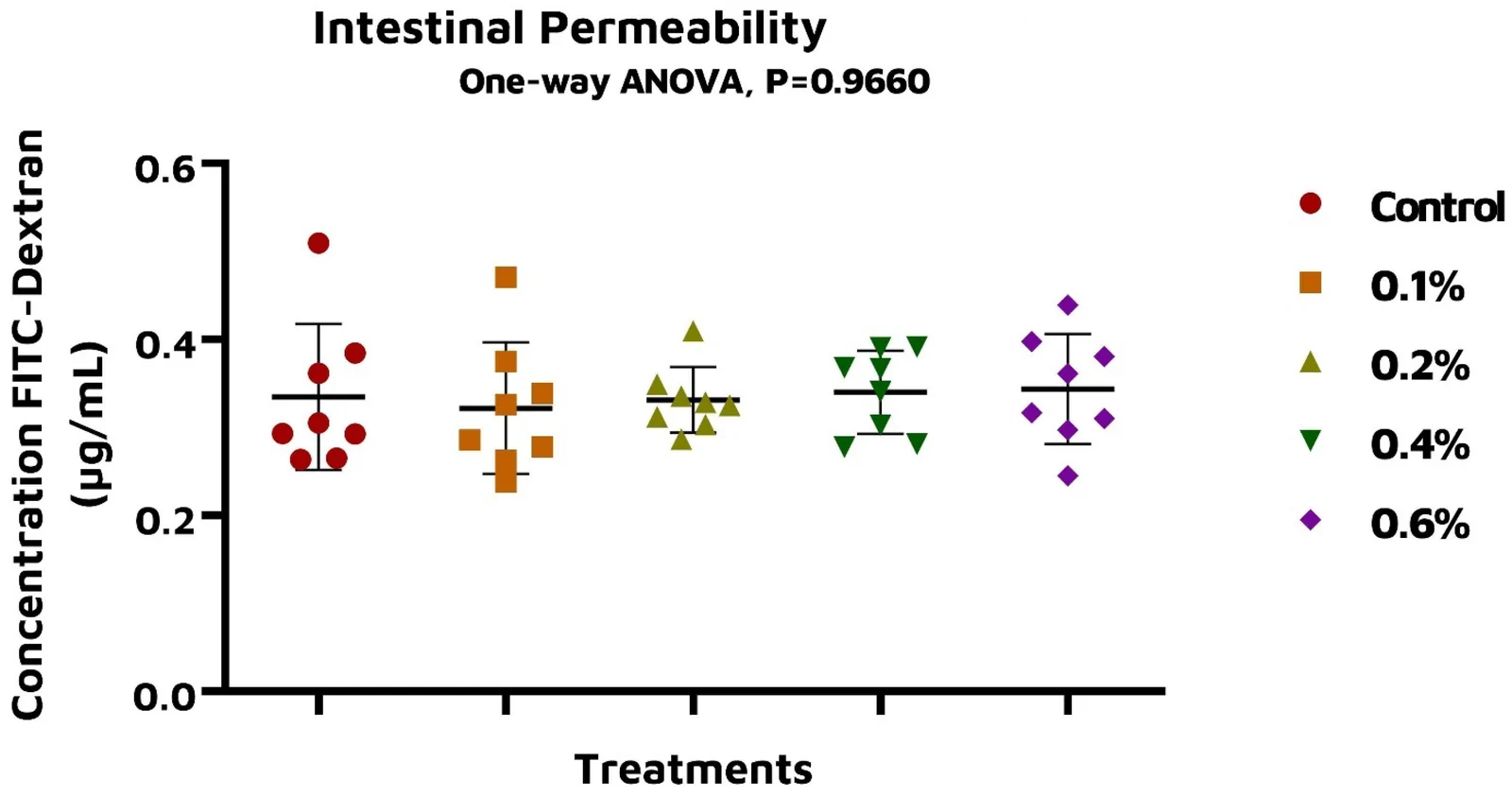
Effect of isoacids on intestinal permeability, assessed by plasma FITC-dextran concentration of commercial male broilers (µg/mL).

The assessment of intestinal permeability via serum FITC-dextran concentration revealed no significant differences (*p* = 0.966) among the experimental treatments (Figure 3). The mean serum concentrations of the macromolecular marker remained remarkably stable and uniform across all groups, strictly clustering around 0.34 ug/mL for all the inclusion levels. The individual data distribution and standard deviations confirmed a homogeneous response, indicating that the baseline was identical regardless of the additive concentration.

### 3.7. Breast Muscle Metabolomics

**Figure 4:**
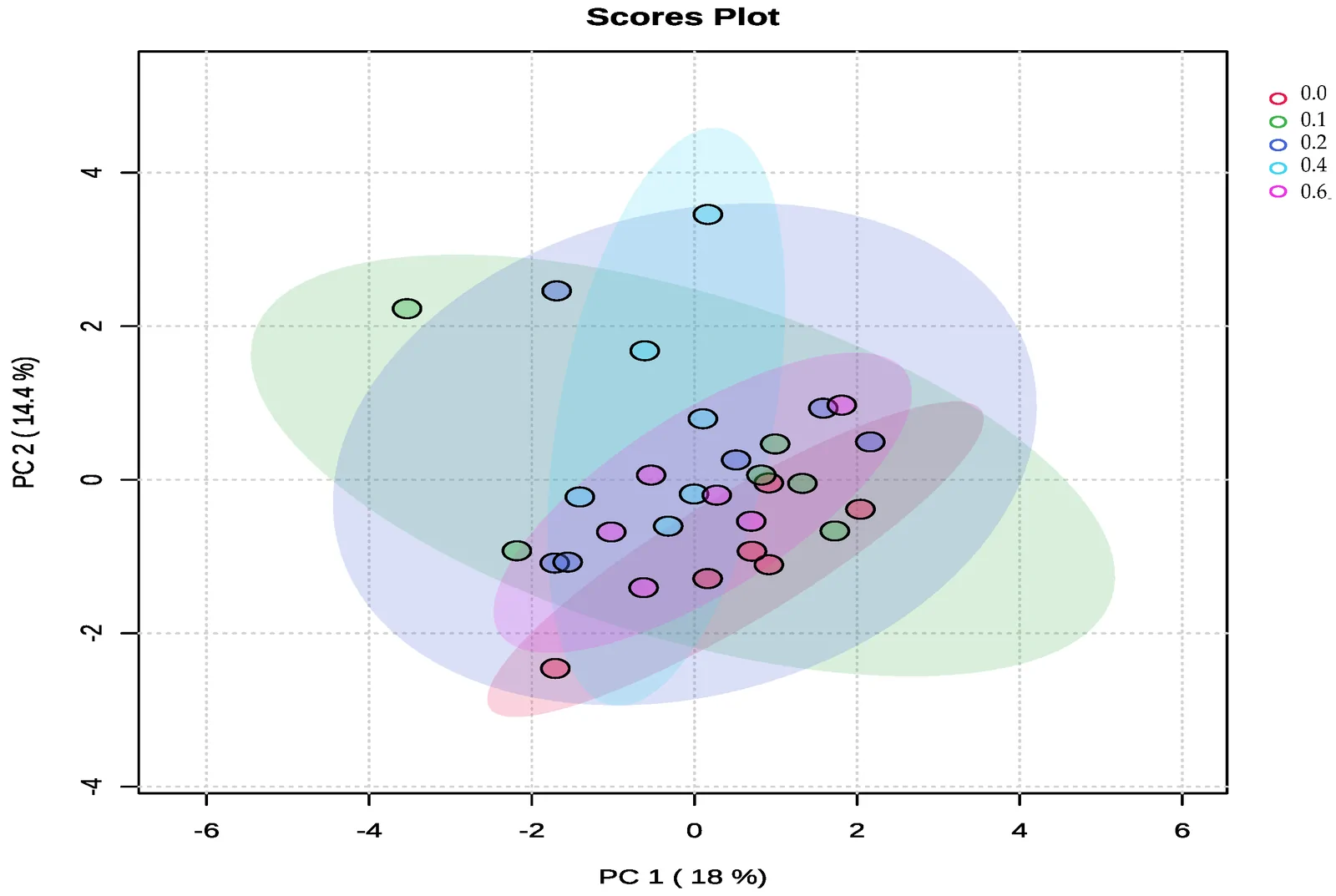
Effect of isoacids on principal component analysis of commercial male broilers.

**Figure 5:**
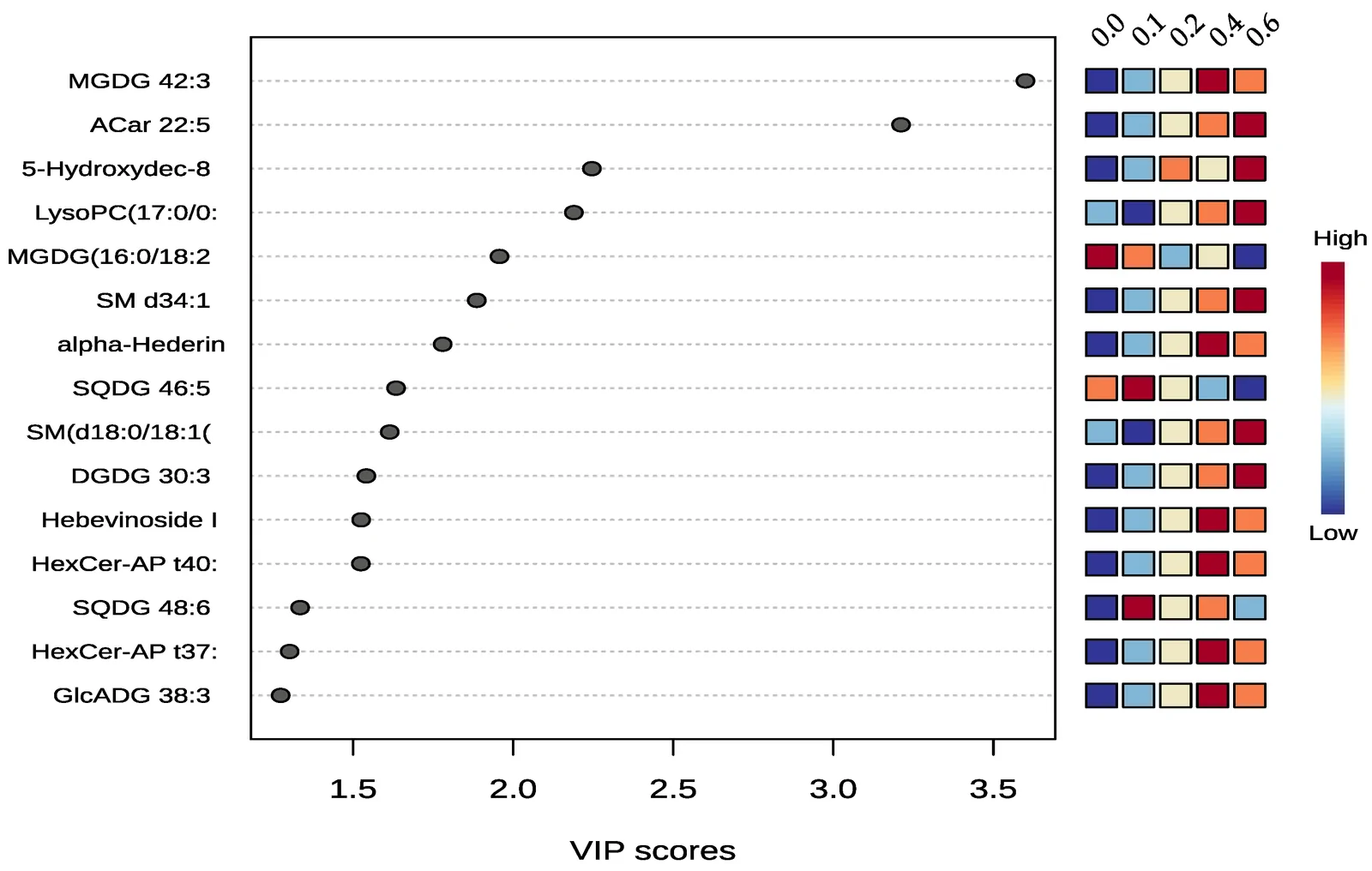
Effect of isoacids on VIP score breast muscle metabolomics of commercial male broilers.

**Figure 6:**
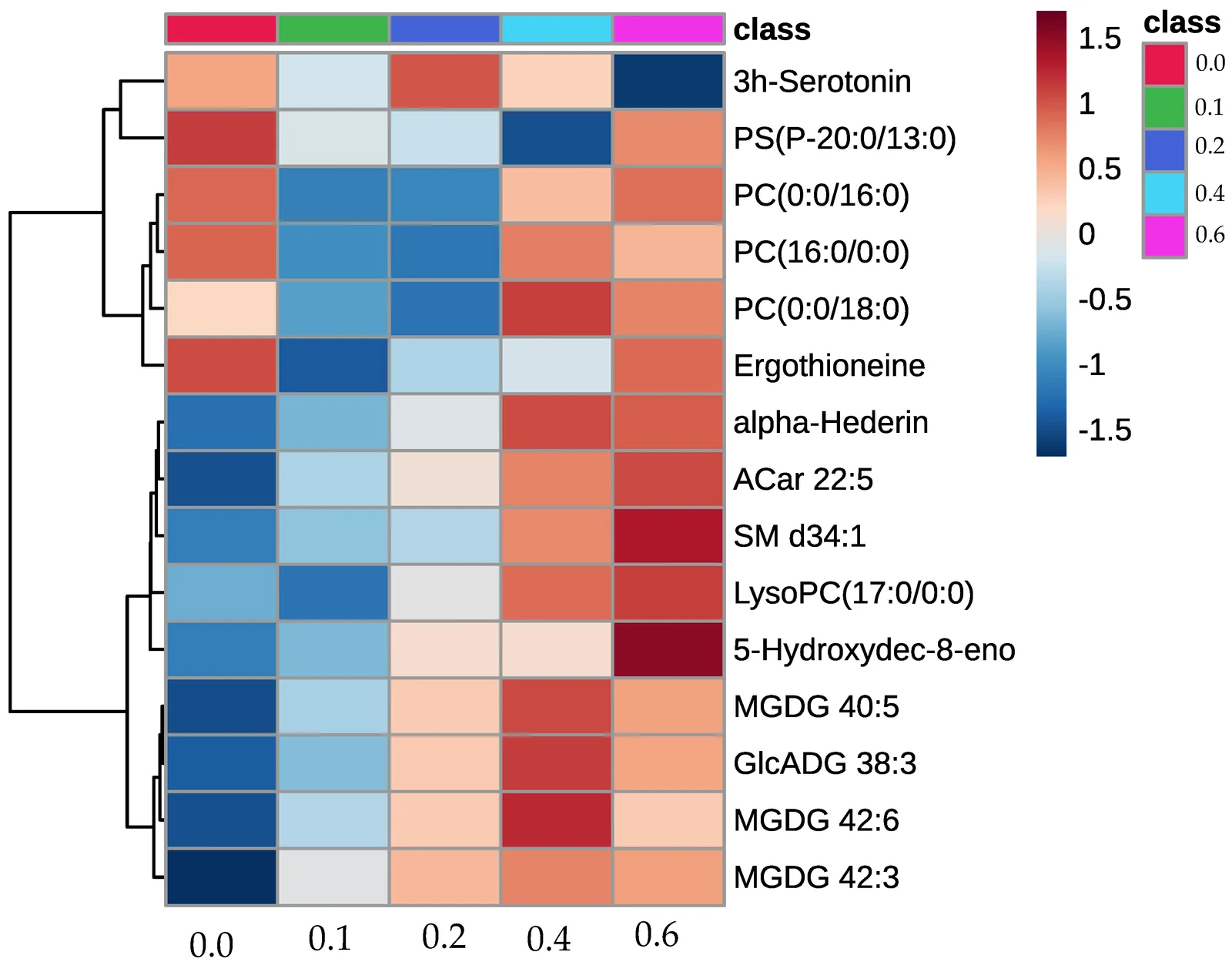
Effect of isoacids on hierarchical clustering discrimination in breast muscle metabolomic analysis of commercial male broilers.

The unsupervised PCA score plot derived from the breast muscle metabolome highlighted a high degree of biological homogeneity among the experimental treatments. As shown in Figure 4, the confidence ellipses for the 0.0% and the 0.1% to 0.6% supplemented groups exhibited substantial spatial overlap along the PC1 (18%) and PC2 (14.4%) axes. This global uniformity suggests that dietary ISO supplementation did not severely disrupt overall metabolic homeostasis. Because PCA did not reveal clear natural clustering, PLS-DA Variable Importance in Projection (VIP) analysis was applied to uncover the specific metabolic shifts induced by the treatments. The PLS-DA VIP analysis was used as an exploratory tool to identify the top 15 discriminant metabolites (Figure 5), which were further resolved via hierarchical clustering into distinct, dose-responsive abundance blocks (Figure 6). The 0.0% group was characterized by an enrichment of structural phospholipids such as PC (0:0/16:0) and PC (16:0/0:0), and the antioxidant ergothioneine (Figure 6). Dietary ISO supplementation modified the relative concentration of the remaining candidate metabolites; specifically, galactolipids like MGDG 42:3, MGDG 42:6, and GlcADG 38:3, along with alpha-Hederin, peaked at the intermediate 0.4% inclusion level (Figure 5 and Figure 6). Conversely, sphingomyelins (SM d34:1), 5-Hydroxydec-8-enoate, LysoPC (17:0/0:0), and the long-chain acylcarnitine ACar 22:5 maximized their relative abundance at the 0.4% and 0.6% dietary doses (Figure 5 and Figure 6). Furthermore, a transient peak for 3 h-Serotonin was recorded at the 0.2% inclusion level (Figure 6). Finally, at the maximum 0.6% dietary dose, a partial numerical reversion toward baseline control levels was observed for specific lipid clusters, particularly galactolipids and alpha-Hederin. The specific biochemical classifications, VIP scores, and physiological response trends of these top discriminant metabolites are detailed in Table 6.

## 4. Discussion

The improvement in body weight gain observed during the early-life phases suggests that dietary ISO enhanced the efficiency of nutrient utilization during the period of greatest metabolic demand. Although the productive advantage was attenuated by the end of the trial, early life improvements are biologically relevant because intestinal development, microbial colonization, and metabolic programming occur predominantly during the first two weeks of life [25]. During these phases, the broiler’s GIT is structurally and enzymatically immature. Exogenous ISO likely provide an easily assimilable energy source for both the gut mucosa and peripheral tissues, bypassing complex metabolic breakdown pathways, allowing a greater proportion of available energy and amino acids to be directed toward productive processes [26]. This early growth advantage was accompanied by reduced abdominal fat deposition. The reduction in abdominal adiposity occurred rapidly at the lowest supplementation level (0.10%) and plateaued thereafter, suggesting that the microbial or metabolic threshold required to downregulate lipid deposition is achieved at relatively low doses of ISO. There is evidence indicating that the intestinal microbiome acts as a critical regulator of lipid metabolism, significantly influencing adiposity and obesity [27].

Dietary ISO supplementation did not affect physicochemical meat quality traits, indicating that muscle properties were preserved across treatments. This is an important finding, as improvements in performance and body composition should not compromise product quality. The linear increase in TBARS values suggests a dose-dependent change in lipid oxidation status. Similar responses have been reported in studies evaluating dietary interventions that modify lipid metabolism and antioxidant balance in poultry muscle [28]. Emerging evidence suggests that gut microbiota may also influence muscle quality traits beyond growth performance, including structural and biochemical characteristics of breast muscle. For instance, alterations in cecal microbiota have been associated with the development of muscle myopathies [29].

ISO supplementation did not significantly alter alpha diversity metrics in either intestinal segment, indicating that microbial richness and overall diversity were maintained despite the dietary intervention. The preservation of alpha diversity suggests that ISO supplementation did not disrupt the ecological stability of the intestinal microbiota. This finding is consistent with the concept that nutritional interventions may modify microbial composition without necessarily affecting ecosystem diversity, particularly when microbial communities possess substantial functional redundancy. Furthermore, the cecum consistently exhibited greater diversity than the ileum, which agrees with previous reports describing the cecum as the most densely colonized and metabolically active microbial compartment in broiler chickens [30].

Beta diversity analyses revealed significant restructuring of microbial communities, particularly within the cecum. These findings indicate that dietary ISO altered how microbial populations were organized within the cecal ecosystem rather than simply changing microbial richness. Because the cecum represents the primary site of microbial fermentation in poultry, its greater responsiveness to dietary modulation was not unexpected and supports previous observations that cecal microbial communities are particularly sensitive to nutritional interventions [3]. The mechanisms underlying microbial restructuring differed between intestinal compartments.

While the cecum exhibited significant responses across abundance based and phylogenetic metrics, the ileum showed significance only for Jaccard and Unweighted UniFrac distances. This pattern suggests that ISO supplementation primarily influenced microbial membership in the ileum, affecting the presence or absence of taxa without substantially altering the abundance of dominant populations. Conversely, cecal communities underwent a broader ecological reorganization involving both community composition and abundance distribution. Such compartment-specific responses are consistent with the distinct physiological roles of the ileum and cecum and have been previously reported in poultry microbiome studies [30].

Taxonomic responses at lower hierarchical levels were relatively modest. The absence of significant changes in the Bacillota/Bacteroidota ratio and the lack of clear clustering patterns in genus-level heatmaps suggest that ISO supplementation did not induce large-scale replacement of dominant microbial groups. Instead, microbial responses appeared to be distributed across multiple taxa. This observation is biologically relevant because functional changes within intestinal ecosystems frequently occur through shifts in community organization rather than dramatic changes in dominant phyla. Among the taxa evaluated, Lachnospiraceae represented the most consistent response to dietary treatment, remaining significant after multiple comparison correction. Members of this family are recognized as important anaerobic fermenters involved in carbohydrate degradation and short-chain fatty acid production within the poultry cecum. Lachnospiraceae are commonly identified among the dominant butyrate-producing bacterial groups in broilers and contribute substantially to fermentative metabolism [31]. Additional responses were detected for Peptostreptococcaceae, Turicibacteraceae, *Romboutsia*, and *Turicibacter* during the initial Kruskal–Wallis analyses; however, these differences did not remain significant after post hoc correction. Therefore, any numerical shifts within these specific taxa must be treated as strictly exploratory observations rather than confirmed treatment related modulation. While our current statistical results cannot assign functional relevance to these unconfirmed taxa, their general roles in amino acid fermentation (e.g., Peptostreptococcaceae, *Romboutsia*) and lipid metabolism (*Turicibacter*) highlight them as interesting targets for future targeted investigations [32].

Cecal SCFA profiles were largely stable across treatments, with acetate remaining the dominant metabolite. In contrast, isobutyric acid exhibited a marginal linear trend (*p* = 0.0443) in response to increasing dietary ISO inclusion, though the overall treatment effect was not significant. Because isobutyrate is produced primarily through microbial fermentation of branched-chain amino acids, these subtle numerical shifts might hint at a potential influence on amino acid fermentation pathways, though this observation should be interpreted with caution given the statistical limitations [7]. While speculative, the numerical trend in cecal isobutyrate, alongside the remodeling of lipid-associated metabolites and reduction in abdominal fat, could suggest a preliminary link between microbial branched-chain amino acid fermentation and host lipid metabolism that warrants further targeted investigation [33]. Dietary ISO supplementation did not compromise gut barrier function under the conditions of this experiment. This is an important finding, as it suggests that performance and body composition responses were achieved without detrimental effects on intestinal integrity. Because the translocation of this high-molecular-weight polymer into systemic circulation occurs exclusively when paracellular pathways are compromised, the lack of significant differences indicates that dietary ISO does not impair intestinal barrier permeability under the conditions evaluated [34]. While our study focused on the cecal environment due to its primary role in avian fermentation, future research should consider mapping VFA and isoacid profiles along the entire gastrointestinal tract, including the upper segments and colon, to fully elucidate their spatial production and absorption dynamics.

Metabolomic analysis of breast muscle revealed a metabolic architecture across treatments, as indicated by the strong overlap observed in PCA space. This suggests that dietary ISO supplementation did not induce a broad scale reprogramming of muscle metabolism but rather maintained overall metabolic homeostasis. A marked dose-dependent, nonlinear response was observed across metabolite clusters. Intermediate inclusion levels (0.4%) were associated with the highest abundance of galactolipids. This specific enrichment indicates a distinct metabolomic shift in the muscle tissue at this inclusion rate, reflecting a targeted accumulation of these plant-derived lipids. This mechanism is consistent with reports by Newman et al. (2002) [35], who noted that avian breast muscle undergoes significant structural remodeling and selective incorporation of dietary fatty acids into cellular phospholipids based on dietary inputs. Another study demonstrated that changing the proportions of plant-derived lipids in broiler diets triggers clear dose-dependent adjustments in the breast muscle lipid profiles, reflecting adaptive limits in how birds deposit dietary lipid fractions into the pectoralis major [36]. The higher inclusion levels (0.6%) were characterized by increased abundance of sphingomyelins, lysophospholipids, and acylcarnitines. This specific upregulation suggests altered lipid handling and transport processes, which could indicate a potential mitochondrial energy challenge (acylcarnitine accumulation) and increased cell membrane turnover (lysophospholipid and sphingomyelin accumulation) [37,38]. The presence of alpha hederin and hebevinenoside among the most discriminant metabolites indicates shifts in plant-derived saponin or glycoside metabolism, possibly influenced by gut microbiota activity. ISO can modulate microbial fermentation, which in turn affects the conversion and absorption of these phytochemical derivatives.

## 5. Conclusions

In conclusion, dietary ISO supplementation represents a viable and effective nutritional strategy to enhance early-life growth performance and modulate body composition in broiler chickens. The inclusion of a 2:1 blend of isobutyrate and 2-methylbutyrate at 0.4% significantly optimized feed conversion efficiency during the critical starter phases (0 to 21 days) and quadratically minimized abdominal fat deposition across an effective inclusion range of 0.10% to 0.40%. Importantly, these phenotypic improvements were achieved without any detrimental effects on gut barrier function or intestinal microbial alpha diversity, and were accompanied by a significant restructuring of the cecal microbial community and a linear increase in cecal isobutyric acid concentration.

Furthermore, breast muscle metabolomics indicated that an inclusion level of 0.4% is characterized by galactolipid enrichment. Conversely, exceeding these levels at 0.6% provides no further productive benefits and triggers a distinct metabolic shift characterized by the accumulation of lysophospholipids and acylcarnitines. Therefore, ISO supplementation within this effective response range offers the poultry industry a sustainable tool to optimize feed efficiency and carcass quality, supporting high standard broiler production without adverse effects on the evaluated performance, carcass, meat quality and intestinal permeability endpoints.

## Figures and Tables

**Table 2:** Effect of isoacids on performance parameters of commercial male broilers.

	Treatments						*p* Values ^3^	
	0.0	0.1	0.2	0.4	0.6	SEM ^2^	Levels	Regression
Variables ^1^								
1 to 7 d								
ADWG, g	20.78 ^ab^	20.08 ^b^	21.24 ^ab^	21.80 ^a^	21.24 ^ab^	0.160	<0.0067	<0.0048 ^L^
ADFI, g	22.72	22.31	21.97	22.34	21.65	0.166	0.3143	NS
FCR, g/g	1.093 ^bc^	1.111 ^c^	1.034 ^ab^	1.024 ^a^	1.019 ^a^	0.009	<0.0006	<0.0001 ^L^
1 to 21 d								
ADWG, g	47.54 ^b^	46.51 ^b^	50.65 ^a^	51.72 ^a^	48.99 ^ab^	0.405	<0.0001	<0.0001 ^L^
ADFI, g	60.28 ^ab^	56.75 ^c^	60.12 ^abc^	62.06 ^a^	58.24 ^bc^	0.457	0.0012	0.0031 ^Q^
FCR, g/g	1.267 ^b^	1.220 ^ab^	1.186 ^a^	1.199 ^a^	1.188 ^a^	0.007	<0.0001	0.0453 ^L^
1 to 35 d								
ADWG, g	65.22	65.60	67.98	69.88	67.80	0.574	0.0576	0.0042 ^L^
ADFI, g	93.67	92.42	94.62	98.95	93.81	0.771	0.0681	0.0169 ^L^
FCR, g/g	1.436	1.408	1.391	1.415	1.383	0.013	0.0814	NS
1 to 42 d								
ADWG, g	71.23	71.98	72.70	72.78	71.91	0.548	0.9060	NS
ADFI, g	104.09	101.40	103.40	106.97	103.11	0.890	0.3939	NS
FCR, g/g	1.461	1.408	1.422	1.469	1.433	0.011	0.3980	NS

^1^ ADWG: average daily weight gain; ADFI: average daily feed intake; FCR: feed conversion ratio; ^L^: linear; ^Q^: quadratic; NS: Not significant (*p* > 0.05). ^2^ SEM: standard error of means. ^3^: Values followed by different letters in the same column differ significantly by Tukey’s test (*p* < 0.05).

**Table 3:** Effect of isoacids on carcass, breast meat, and cut-up part yields of commercial male broilers.

	Parameters				
Levels	Carcass (%)	Breast (%)	Legs (%)	Wings (%)	Abd Fat (%)
0.0	75.68	33.39	30.91	10.54	1.57 ^a^
0.1	75.19	32.84	31.17	10.49	1.04 ^b^
0.2	75.31	34.11	30.35	10.59	1.00 ^b^
0.4	76.12	34.11	30.06	10.30	1.13 ^b^
0.6	75.56	33.95	30.47	10.38	1.02 ^b^
SEM ^1^	0.183	0.204	0.135	0.047	0.037
Probabilities ^2^					
Levels	0.5339	0.2052	0.0615	0.2751	<0.0001
Regression ^3^	NS	NS	0.0111 ^L^	NS	0.0001 ^Q^

^1^ SEM: standard error of means. ^2^ Values followed by different letters in the same column differ significantly by Tukey’s test (*p* < 0.05). ^3 L^: Linear. ^Q^: Quadratic. NS: Not significant (*p* > 0.05).

**Table 4:** Effect of isoacids on Beta diversity analysis in cecum and ileum of commercial male broilers.

Distance Method	Cecal Content		Ileum Content	
	R^2^	Pr (>F)	R^2^	Pr (>F)
Bray–Curtis	0.1713	0.0213 (*)	0.1355	0.4508
Jaccard	0.1567	0.0161 (*)	0.1715	0.0104 (*)
Unweighted Unifrac	0.1507	0.1224	0.1677	0.0145
Weighted Unifrac	0.1138	0.0312 (*)	0.1306	0.4625

* means statistical difference.

**Table 5:** Effect of isoacids on the concentration of short-chain fatty acids in cecal contents of commercial male broilers (%).

	% of Total SCFAs					
Levels	Acetic	Propionic	Isobutyric	Butyric	Isovaleric	Valeric
0.0	70.65	9.05	1.06	16.48	0.73	1.47
0.1	72.43	10.10	1.45	13.36	1.16	1.49
0.2	73.80	8.05	1.32	14.60	0.95	1.30
0.4	70.39	9.93	1.55	15.79	1.10	1.25
0.6	73.34	10.03	1.45	12.56	1.33	1.29
SEM ^1^	0.678	0.369	0.064	0.773	0.092	0.055
Probabilities						
Levels	0.4051	0.3459	0.165	0.4929	0.3329	0.5364
Regression ^2^	NS	NS	0.0443 ^L^	NS	NS	NS

^1^ SEM: standard error of means. ^2 L^: Linear. NS: Not significant (*p* > 0.05).

**Table 6:** Key discriminant metabolites identified in broiler breast muscle following dietary ISO supplementation, ranked by Variable Importance in Projection (VIP) score.

Metabolite	Biochemical Classification/Pathway	VIP Score	Response Across Treatments
MGDG 42:3	Galactolipid/Membrane Lipid	3.6	Upregulated at intermediate levels (0.2–0.4%)
ACar 22:5	Acylcarnitine/Fatty Acid Oxidation	3.2	Upregulated at intermediate to high levels (0.4–0.6%)
5-Hydroxydec-8-enoate	Lipid/Fatty Acid Derivative	2.2	Maximized relative abundance at 0.4% and 0.6% doses
LysoPC (17:0/0:0)	Lysophosphatidylcholine/Lipid Transport	2.1	Increased relative abundance at 0.4% and 0.6% doses
MGDG (16:0/18:2)	Galactolipid/Membrane Lipid	1.9	Modulated by ISO supplementation
SM d34:1	Sphingomyelin/Cellular Membrane	1.8	Maximized relative abundance at 0.4% and 0.6% doses
alpha-Hederin	Phytochemical/Saponin Derivative	1.7	Peaked at the intermediate 0.4% inclusion level
SQDG 46:5	Sulfolipid/Membrane Integrity	1.6	Coordinated response with energy pathways
SM (d18:0/18:1)	Sphingomyelin/Cellular Membrane	1.6	Higher abundance correlated with lipid remodeling
DGDG 30:3	Digalactosyldiacylglycerol/Lipid Metabolism	1.5	Stable/Upregulated with isoacid supplementation
Hebevinenoside I	Glycoside Derivative	1.5	Altered via gut microbiota biotransformation
HexCer-AP t40	Hexosylceramide/Sphingolipid Metabolism	1.5	Reflects cellular turnover and membrane remodeling
SQDG 48:6	Sulfolipid/Membrane Integrity	1.3	Coordinated response with energy pathways
HexCer-AP t37	Hexosylceramide/Sphingolipid Metabolism	1.3	Reflects cellular turnover and membrane remodeling
GlcADG 38:3	Glycosyldiacylglycerol/Membrane Lipid	1.3	Peaked at the intermediate 0.4% inclusion level

## Data Availability

The data presented in this study are available upon request from the corresponding author.

## References

[1] Allison M.J., Bryant M.P. (1963). Biosynthesis of branched-chain amino acids from branched-chain fatty acids by rumen bacteria. Arch. Biochem. Biophys..

[2] Andries J.I., Buysse F.X., De Brabander D.L., Cottyn B.G. (1987). Isoacids in ruminant nutrition: Their role in ruminal and intermediary metabolism and possible influences on performances—A review. Anim. Feed Sci. Technol..

[3] Apajalahti J., Vienola K. (2016). Interaction between chicken intestinal microbiota and protein digestion. Anim. Feed Sci. Technol..

[4] Oakley B.B., Lillehoj H.S., Kogut M.H., Kim W.K., Maurer J.J., Pedroso A., Lee M.D., Collett S.R., Johnson T.J., Cox N.A. (2014). The chicken gastrointestinal microbiome. FEMS Microbiol. Lett..

[5] Bedford A., Gong J. (2017). Implications of butyrate and its derivatives for gut health and animal production. Anim. Nutr..

[6] Onrust L., Ducatelle R., Van Driessche K., De Maesschalck C., Vermeulen K., Haesebrouck F., Eeckhaut V., Van Immerseel F. (2015). Steering Endogenous Butyrate Production in the Intestinal Tract of Broilers as a Tool to Improve Gut Health. Front. Vet. Sci..

[7] Windey K., de Preter V., Verbeke K. (2012). Relevance of protein fermentation to gut health. Mol. Nutr. Food Res..

[8] Pieper R., Villodre Tudela C., Taciak M., Bindelle J., Pérez J.F., Zentek J. (2016). Health relevance of intestinal protein fermentation in young pigs. Anim. Health Res. Rev..

[9] Cave N.A. (1984). Effect of Dietary Propionic and Lactic Acids on Feed Intake by Chicks. Poult. Sci..

[10] Pinchasov Y., Jensen L.S. (1989). Effect of Short-Chain Fatty Acids on Voluntary Feed of Broiler Chicks. Poult. Sci..

[11] Suiryanrayna M.V.A.N., Ramana J.V. (2015). A review of the effects of dietary organic acids fed to swine. J. Anim. Sci. Biotechnol..

[12] Cobb Vantress (2021). Cobb 500 Broiler Management Guide. https://www.cobbgenetics.com/.

[13] Rostagno H.S., Albino L.T., Calderano A.A., Hannas M.I., Sakomura N.K., Perazzo F.G., Rocha G.G., Saraiva A., Texeira M.L., Genova J.L. (2024). Tabelas Brasileiras para aves e Suínos: Composição de Alimentos e Exigências Nutricionais.

[14] Cobb Vantress (2022). Cobb 500 Broiler-Performance & Nutrition Supplement (2022). https://www.cobbgenetics.com/.

[15] Abudabos A.M., Suliman G.M., Al-Owaimer A.N., Al Sulaiman A.R., Alharthi A.S. (2021). Effects of Nano Emulsified Vegetable Oil and Betaine on Growth Traits and Meat Characteristics of Broiler Chickens Reared under Cyclic Heat Stress. Animals.

[16] Da Silva-Buzanello R.A., Schuch A.F., Gasparin A.W., Torquato A.S., Scremin F.R., Canan C., Soares A.L. (2019). Quality parameters of chicken breast meat affected by carcass scalding conditions. Asian-Australas. J. Anim. Sci..

[17] Mohd Shaufi M.A., Sieo C.C., Chong C.W., Gan H.M., Ho Y.W. (2015). Deciphering chicken gut microbial dynamics based on high-throughput 16S rRNA metagenomics analyses. Gut Pathog..

[18] Callahan B.J., McMurdie P.J., Holmes S.P. (2017). Exact sequence variants should replace operational taxonomic units in marker-gene data analysis. ISME J..

[19] Erwin E.S., Marco G.J., Emery E.M. (1961). Volatile Fatty Acid Analyses of Blood and Rumen Fluid by Gas Chromatography. J. Dairy Sci..

[20] Liu J., Teng P.Y., Kim W.K., Applegate T.J. (2021). Assay considerations for fluorescein isothiocyanate-dextran (FITC-d): An indicator of intestinal permeability in broiler chickens. Poult. Sci..

[21] Want E.J., O’Maille G., Smith C.A., Brandon T.R., Uritboonthai W., Qin C., Trauger S.A., Siuzdak G. (2006). Solvent-Dependent Metabolite Distribution, Clustering, and Protein Extraction for Serum Profiling with Mass Spectrometry. Anal. Chem..

[22] Hemmer S., Manier S.K., Fischmann S., Westphal F., Wagmann L., Meyer M.R. (2020). Comparison of Three Untargeted Data Processing Workflows for Evaluating LC-HRMS Metabolomics Data. Metabolites.

[23] Parks D.H., Chuvochina M., Rinke C., Mussig A.J., Chaumeil P.A., Hugenholtz P. (2022). GTDB: An ongoing census of bacterial and archaeal diversity through a phylogenetically consistent, rank normalized and complete genome-based taxonomy. Nucleic Acids Res..

[24] Pang Z., Chong J., Zhou G., De Lima Morais D.A., Chang L., Barrette M., Gauthier C., Jacques P.-É., Li S., Xia J. (2021). MetaboAnalyst 5.0: Narrowing the gap between raw spectra and functional insights. Nucleic Acids Res..

[25] Proszkowiec-Weglarz M., Miska K.B., Ellestad L.E., Schreier L.L., Kahl S., Darwish N., Campos P., Shao J. (2022). Delayed access to feed early post-hatch affects the development and maturation of gastrointestinal tract microbiota in broiler chickens. BMC Microbiol..

[26] Gilbert M.S., Ijssennagger N., Kies A.K., van Mil S.W.C. (2018). Protein fermentation in the gut; implications for intestinal dysfunction in humans, pigs, and poultry. Am. J. Physiol.-Gastrointest. Liver Physiol..

[27] Xiang H., Gan J., Zeng D., Li J., Yu H., Zhao H., Yang Y., Tan S., Li G., Luo C. (2021). Specific Microbial Taxa and Functional Capacity Contribute to Chicken Abdominal Fat Deposition. Front. Microbiol..

[28] Long X., Zhang F., Wang L., Wang Z. (2024). The chicken cecal microbiome alters bile acids and riboflavin metabolism that correlate with intramuscular fat content. Front. Microbiol..

[29] Zhang X., Zhang L., Li X., White S., Campbell Y.L., Ruiz A.A., To K.V., Zhai W., Schilling M.W. (2021). Cecal microbiota contribute to the development of woody breast myopathy. Poult. Sci..

[30] Liao X., Shao Y., Sun G., Yang Y., Zhang L., Guo Y., Luo X., Lu L. (2020). The relationship among gut microbiota, short-chain fatty acids, and intestinal morphology of growing and healthy broilers. Poult. Sci..

[31] Borda-Molina D., Seifert J., Camarinha-Silva A. (2018). Current Perspectives of the Chicken Gastrointestinal Tract and Its Microbiome. Comput. Struct. Biotechnol. J..

[32] Lynch J.B., Gonzalez E.L., Choy K., Faull K.F., Jewell T., Arellano A., Liang J., Yu K.B., Paramo J., Hsiao E.Y. (2023). Gut microbiota Turicibacter strains differentially modify bile acids and host lipids. Nat. Commun..

[33] Bai J., Greene E., Li W., Kidd M.T., Dridi S. (2015). Branched-chain amino acids modulate the expression of hepatic fatty acid metabolism-related genes in female broiler chickens. Mol. Nutr. Food Res..

[34] Awad W.A., Ruhnau D., Hess C., Doupovec B., Schatzmayr D., Hess M. (2019). Feeding of deoxynivalenol increases the intestinal paracellular permeability of broiler chickens. Arch. Toxicol..

[35] Newman R.E., Bryden W.L., Fleck E., Ashes J.R., Buttemer W.A., Storlien L.H., Downing J.A. (2002). Dietary n-3 and n-6 fatty acids alter avian metabolism: Metabolism and abdominal fat deposition. Br. J. Nutr..

[36] Abdulla N.R., Loh T.C., Foo H.L., Alshelmani M.I., Akit H. (2019). Influence of Dietary Ratios of n-6: n-3 Fatty Acid on Gene Expression, Fatty Acid Profile in Liver and Breast Muscle Tissues, Serum Lipid Profile, and Immunoglobulin in Broiler Chickens. J. Appl. Poult. Res..

[37] Abasht B., Mutryn M.F., Michalek R.D., Lee W.R. (2016). Oxidative Stress and Metabolic Perturbations in Wooden Breast Disorder in Chickens. PLoS ONE.

[38] Liu R., Kong F., Xing S., He Z., Bai L., Sun J., Tan X., Zhao D., Zhao G., Wen J. (2022). Dominant changes in the breast muscle lipid profiles of broiler chickens with wooden breast syndrome revealed by lipidomics analyses. J. Anim. Sci. Biotechnol..

